# The Speech-to-Song Illusion Is Reduced in Speakers of Tonal (vs. Non-Tonal) Languages

**DOI:** 10.3389/fpsyg.2016.00662

**Published:** 2016-05-09

**Authors:** Kankamol Jaisin, Rapeepong Suphanchaimat, Mauricio A. Figueroa Candia, Jason D. Warren

**Affiliations:** ^1^Dementia Research Centre, UCL Institute of Neurology, University College LondonLondon, UK; ^2^Department of Psychiatry, Faculty of Medicine, Thammasat UniversityBangkok, Thailand; ^3^Department of Global Health and Development, London School of Hygiene and Tropical MedicineLondon, UK; ^4^International Health Policy Program, Ministry of Public HealthBangkok, Thailand; ^5^Department of Speech, Hearing and Phonetic Sciences, Faculty of Brain Sciences, University College LondonLondon, UK

**Keywords:** speech-to-song illusion, tonal language, bilingual, prosody, music

## Abstract

The speech-to-song illusion has attracted interest as a probe of the perceptual interface between language and music. One might anticipate differential speech-to-song effects in tonal vs. non-tonal languages, since these language classes differ importantly in the linguistic value they assign to tones. Here we addressed this issue for the first time in a cohort of 20 healthy younger adults whose native language was either tonal (Thai, Mandarin) or non-tonal (German, Italian) and all of whom were also fluent in English. All participants were assessed using a protocol designed to induce the speech-to-song illusion on speech excerpts presented in each of the five study languages. Over the combined participant group, there was evidence of a speech-to-song illusion effect for all language stimuli and the extent to which individual participants rated stimuli as “song-like” at baseline was significantly positively correlated with the strength of the speech-to-song effect. However, tonal and non-tonal language stimuli elicited comparable speech-to-song effects and no acoustic language parameter was found to predict the effect. Examining the effect of the listener's native language, tonal language native speakers experienced significantly weaker speech-to-song effects than non-tonal native speakers across languages. Both non-tonal native language and inability to understand the stimulus language significantly predicted the speech-to-song illusion. These findings together suggest that relative propensity to perceive prosodic structures as inherently linguistic vs. musical may modulate the speech-to-song illusion.

## Introduction

Speech and song constitute complex pitch and rhythmic patterns: these patterns have distinctive acoustic characteristics but engage shared perceptual mechanisms and brain circuitry (Merrill et al., 2012; Hausen et al., 2013). A striking example of the blurring of cognitive boundaries between speech and song is the speech-to-song effect: an illusory perceptual transformation whereby a spoken phrase comes spontaneously to be perceived as sung following repetition. Since its first description by Deutsch (2003), this illusion has attracted interest as a probe of the perceptual boundaries between speech and music, the salience and stability of pitch percepts and auditory perceptual ambiguity (Deutsch et al., 2011; Tierney et al., 2013). The illusion is likely to arise from modulation of responses in temporo-parieto-frontal circuitry mediating pitch coding and production (Tierney et al., 2013); induction of the effect depends both on relative stability of pitch (fundamental frequency) contours within syllables and the precise ordering of syllables during repetition (Zhang, 2010; Deutsch et al., 2011; Falk et al., 2014), suggesting that the illusion involves integration of musical pitch characteristics inherent in prosodic speech streams. However, the strength of the speech-to-song illusion is enhanced if the source language is relatively more difficult for the listener to pronounce (Margulis et al., 2015), further suggesting that the effect may also depend on the extent to which language circuitry is “captured” by the auditory speech stream and therefore potentially by previous linguistic experience and perceptual competence (Falk et al., 2014).

Tonal languages present an interesting test case for interpreting the mechanism of the speech-to-song illusion. In these languages (exemplified by Thai and Mandarin Chinese), the pitch of syllables carries linguistic semantic value and has been shown to be processed by dominant hemisphere language networks (in contrast to pitch information in languages that the listener does not understand, which is processed by right hemispheric or non-lateralized networks: Zatorre and Gandour, 2008). The discrete pitch values (corresponding to phonological categories) of tonal languages tend to align pitch patterns in such languages closely with those of music (Liu et al., 2013). However, tonal and non-tonal language native speakers show distinct profiles of lexical pitch processing and tonal language experience is associated with stronger categorical encoding of linguistic pitch patterns (Bidelman and Lee, 2015). To the extent that the speech-to-song illusion is driven by prosodic properties inherent in the speech stream, one might anticipate that tonal language stimuli and non-tonal language stimuli should differ in their propensity to induce the illusion across listeners with different native languages. On the other hand, to the extent the effect is inhibited by linguistic mechanisms “capturing” speech prosody, then the effect should be relatively attenuated by prior familiarity with a tonal language (i.e., reduced in tonal compared with non-tonal native language speakers). While the speech-to-song illusion is well documented in non-tonal languages other than English (Falk et al., 2014; Margulis et al., 2015) and conditions to induce the effect in a tonal language (Mandarin) have been described (Zhang, 2010), little information is presently available concerning the relative strength of the speech-to-song illusion experienced by tonal vs. non-tonal language speakers.

In this study, our primary aim was to determine whether native language tonality (whether one's native language is tonal or non-tonal) influences perception of the speech-to-song illusion. We investigated a cohort of normal young adults who spoke either a tonal language (Thai or Mandarin) or a non-tonal language (Italian or German) as their native tongue and who were all also fluent in English. The speech-to-song illusion was assessed on stimuli presented in each of these languages in all participants: by crossing the factors of native language and stimulus language, we intended this experimental design to allow us to disambiguate effects of native language from acoustic language properties. We hypothesized that the speech-to-song effect would be attenuated across stimulus languages for tonal compared with non-tonal language native speakers, since semantic language mechanisms would dominate perception of prosodic features in tonal native language speakers (Margulis et al., 2015). Extending this hypothesis, we further predicted an enhanced speech-to-song effect for languages that the speaker failed to understand. Inclusion of bilingual participants here allowed us to assess the effect in the setting of demonstrated linguistic proficiency and experience across languages, while inclusion of a language in which all participants were proficient (English) provided a reference for interpreting any apparently language-specific perceptual effects.

## Materials and methods

### Participants

Twenty healthy right-handed younger adults (13 female, seven male; aged 20–43 years, mean age 29.7 years) participated. All participants spoke English as their second language; their native language was either primarily tonal (Thai, Mandarin) or primarily non-tonal (German, Italian). As a criterion of entry, participants demonstrated proficiency in English as a second language, based on completion of a minimum of twelve months in university education in an English speaking country, a minimum of eighteen months working in an English speaking workplace and/or superior performance on a standard English language test (e.g., International English Language Testing System (IELTS) score >6.5, Test of English as a Foreign Language—Internet-based Test (TOEFL-IBT) score >79). No participant understood any of the other non-native languages presented in the study. All participants were university staff or students. None had a history of neurological or hearing impairment, language disorders or learning disability; participants varied widely in musical training (ranging from none to over 10 years of formal training on an instrument, tonal language speakers having been exposed to both Western and non-Western musical traditions) but according to participants' self reports, none had congenital amusia or possessed absolute pitch. All were previously naïve to the speech-to-song illusion.

The study was approved by the Local Research Ethics Committee of University College London and all participants gave written informed consent in accordance with the Declaration of Helsinki.

### Experimental stimuli

**Figure 1:**
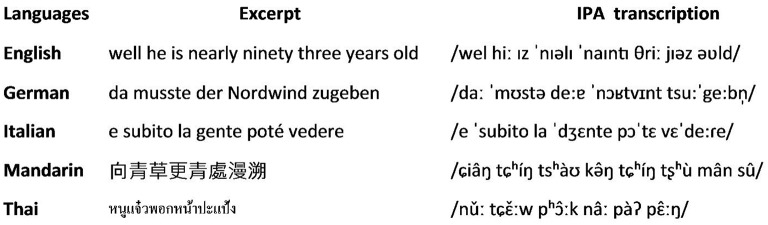
**Language excerpts used as target stimuli**. IPA, International Phonetic Alphabet. Source text passages were as follows: English, the *Grandfather* passage (https://www.d.umn.edu/~cspiller/readingpassages.html); German, the *Nordwind und Sonne* passage (http://www2.ims.uni-stuttgart.de/sgtutorial/nordwind.html); Italian, passage from *Giacomo di cristallo* (http://www.sestogiorno.it/racconti/GiacomodicristalloRodari.pdf); Mandarin, passage created by Yu Ting Huang based on sentences used in Chinese speech therapy practice to expose speech errors; Thai, passage from Sindermsuk (1986).

A standard reading passage used in speech and language studies was recorded in each of the languages spoken by the participants (one passage in Thai, Mandarin, German, Italian, and the English “Grandfather passage”), by female native speakers. A short excerpt was selected from each recording, with duration (mean 2.4 (± 0.4) s) and number of words (between 6 and 10) similar to those shown previously to be effective in generating the speech-to-song illusion in native English speakers (Deutsch, 2003); the excerpts chosen all induced the speech-to-song illusion in pilot testing of graduate student English native language speakers in our laboratory. All excerpts are presented and source passages are referenced in Figure 1; stimulus characteristics (extracted post hoc following the method of Tierney et al., 2013) are summarized in Table S1 and further details about the stimulus analysis procedure and the stimulus sound files are provided as Supplementary Material on-line. Two acoustic parameters, identified as potentially relevant to the speech-to-song illusion in previous work (Tierney et al., 2013), were derived: these parameters captured language stimulus properties of fundamental frequency variability (median absolute deviation of intra-syllable fundamental frequency change) and temporal variability (median absolute deviation of inter-syllable duration change). The selected excerpts were used to induce the speech-to-song effect and are henceforth here designated “target” stimuli. In addition, in order to assess each participant's ability to recognize actual singing in a non-native language, a second excerpt in English from the Grandfather Passage was recorded as a sung phrase by the same female native speaker (using ascending—descending note rows of a major scale). Thus, six stimuli in all (five language test excerpts and the English sung control excerpt) were presented to each participant (in using a restricted stimulus set, we aimed to reduce any overall nonspecific facilitation of the speech-to-song effect).

### Procedure

Stimuli were administered to participants from a notebook computer via headphones at a comfortable listening level in a quiet room. All participants heard all stimuli, presented for each language in “baseline” “induction” and “test” phases. In the initial “baseline” phase, participants were asked to rate each target stimulus on a Likert scale between 0 (“sounds exactly like speech”) and 5 (“sounds exactly like singing”). In the ensuing “induction phase,” 10 repetitions were then presented for each target stimulus; this number of repetitions is in line with procedures shown previously reliably to induce the illusion in native English speakers (Deutsch, 2003). In the subsequent “test” phase, the target stimulus was then presented again embedded in the spoken source passage; the participant's task was to indicate as soon as they heard singing and to rate this for intensity on the same (0–5 point) scale (a value of 0 was here assigned post hoc if the participant did not report hearing singing at all for that trial). Languages were presented in randomized order across the participant group. Finally, the sung English except was presented for rating. Participant responses were recorded for offline analysis.

### Analysis

Participant data were in general not normally distributed and were therefore analyzed using non-parametric or semi-parametric tests. Participant groups (age, gender, education, formal music training) were compared using Kruskal-Wallis and Fisher's exact tests. Illusion rating scores between the baseline and test phases (pre- and post-induction phase) and within and between language groups were compared using Wilcoxon Signed Rank and Mann-Whitney tests. For our purpose here, the speech-to-song illusion effect was defined operationally as any increase in post-induction rating score compared with the pre-induction rating score for that language and participant. The effect of candidate predictors of the speech-to-song effect was assessed first in a bivariate analysis, incorporating the magnitude of the illusion rating as the outcome variable and participant native language (classified as tone or non-tonal), other participant characteristics (age, gender, education, formal music training, ability to understand stimulus language) and stimulus language as predictor variables. Chi-square, Fisher's exact and Ranksum tests were used to identify those predictor variables showing a significant association with the speech-to-song illusion effect. The influence of these variables on the illusion was then compared using multivariate logit regression, in order to assess the effect of each variable taking other variables into account. Two multivariate regression models were used to assess candidate predictor variables: a model with robust standard error and a random effects model (since each participant participated in five experimental rounds, one for each target stimulus/language, both within- and between-individual effects were considered likely a priori to affect the precision of the results). A threshold of *p* < 0.05 was accepted as the criterion of statistical significance for all tests.

## Results

Participant characteristics (grouped by native language) are summarized in Table 1. Participant language groups did not differ significantly in gender distribution, or prior musical training; Thai native speakers were significantly older (Kruskal-Wallis *p* = 0.027) and had significantly more years of education (Kruskal-Wallis *p* = 0.035) than other language groups. All participants recognized stimuli sung in English as sung, as indexed by “song-like” ratings (range 3–5) of these stimuli by each participant and confirming that all participants normally perceived singing in a non-native language.

**Table 1:** **Summary of participant characteristics for combined group and each native language group**.

**Characteristic**	**All (*n* = 20)**	**German (*n* = 5)**	**Italian (*n* = 5)**	**Mandarin (*n* = 5)**	**Thai (*n* = 5)**
Age (years)	29.2 (7.3)	26.0 (1.1)	28.8 (3.0)	28.4 (6.8)	34.5 (2.2)^*^
Gender (male: female)	7:13	3:2	2:3	1:4	1:4
Education (years)	20 (5.0)	18 (0.0)	20 (1.0)	20 (5.0)	24 (3.0)^*^
Formal music training (years)	4.5 (9)	5.0 (4)	5 (10)	0.2 (12)	2 (8)

*Median (inter-quartile range) data are shown*.

* *statistically greater than other groups, p < 0.05*.

**Figure 2:**
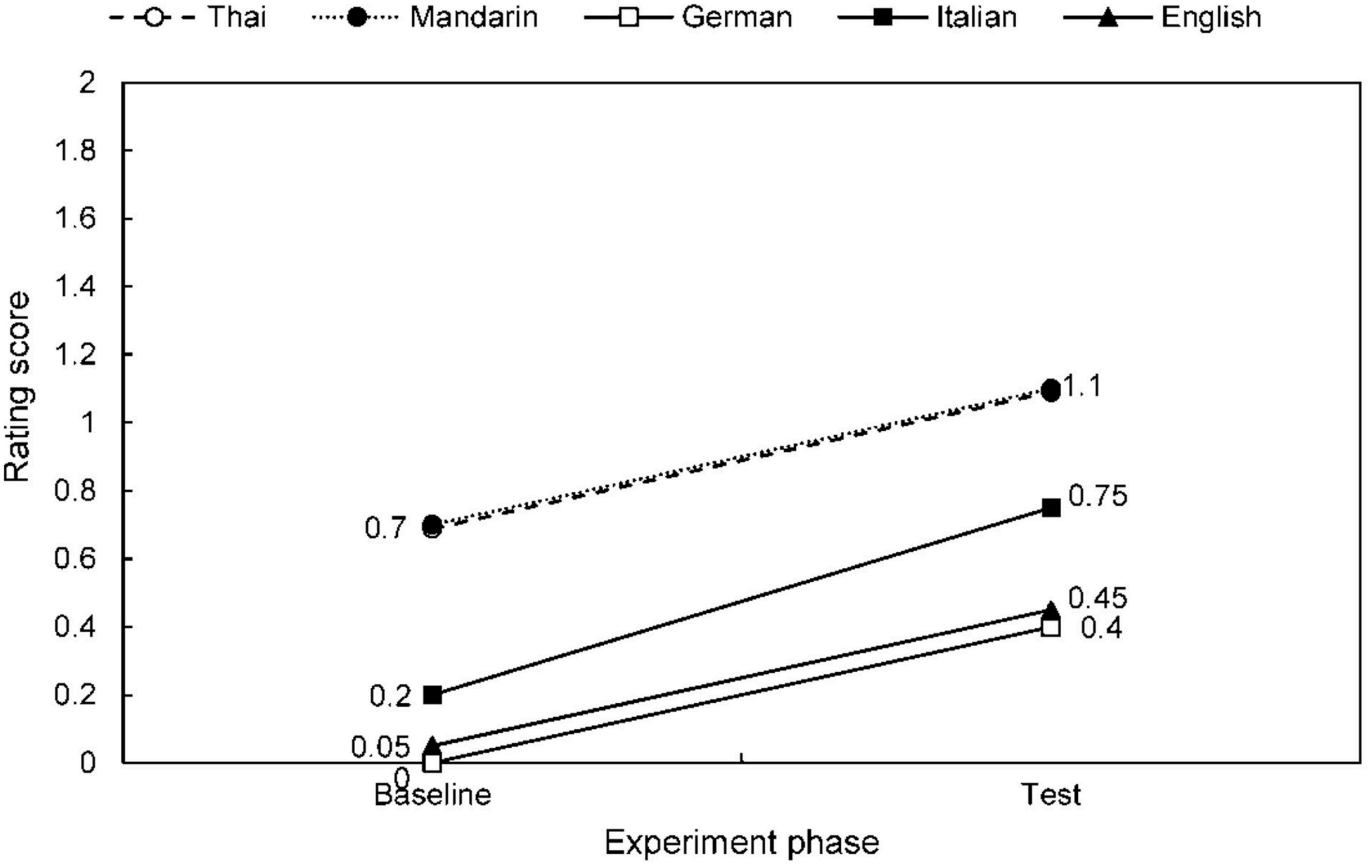
**Mean speech-to-song effect ratings for target stimuli baseline and test phase, for each stimulus language**. Results for the combined participant group are shown (note data for Thai and Mandarin superimposed) see also Table 2.

**Figure 3:**
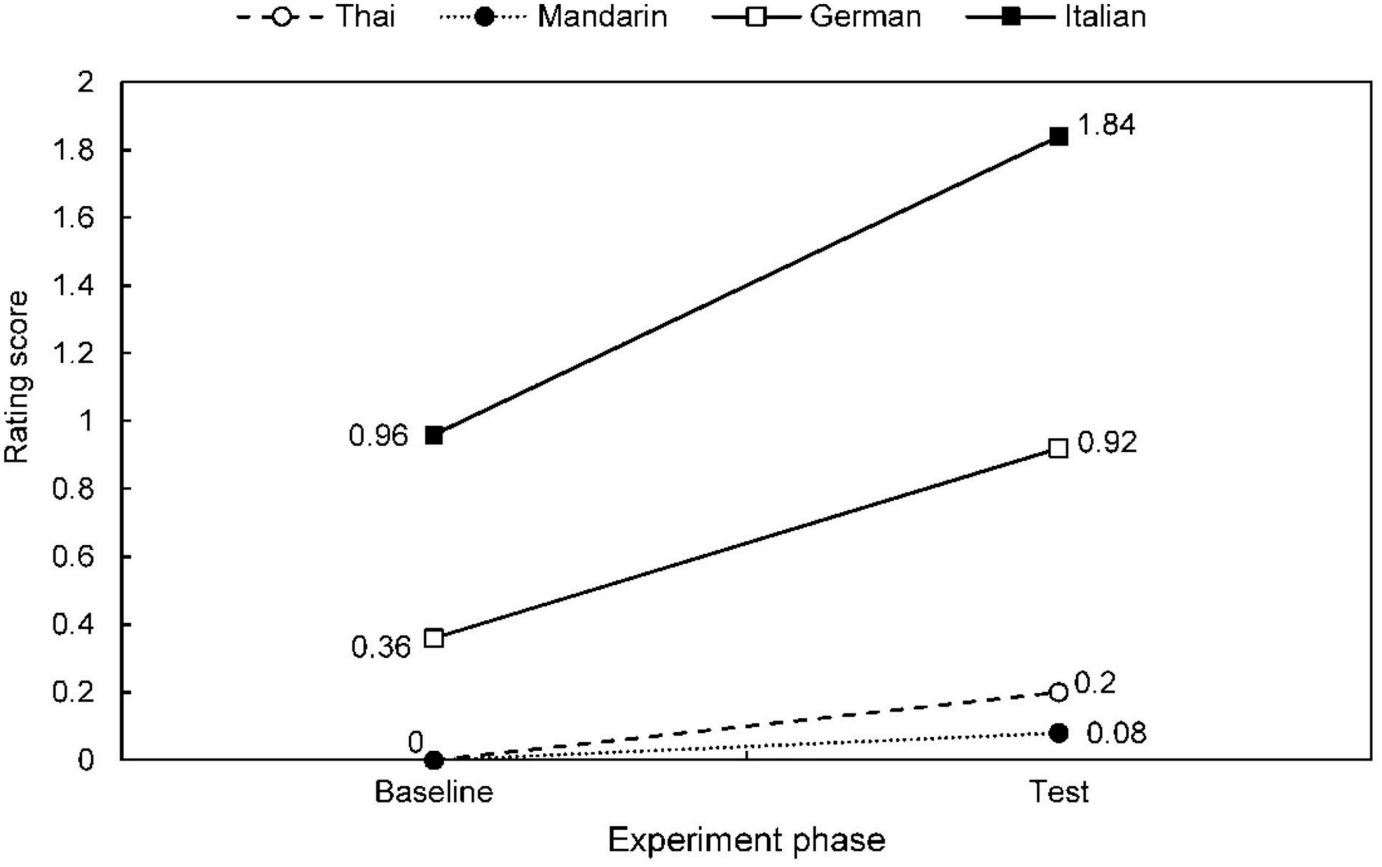
**Mean speech-to-song effect ratings for target stimuli baseline and test phase, for each native language participant group**. Results for all target stimuli combined across languages are shown see also Table 3.

**Figure 4:**
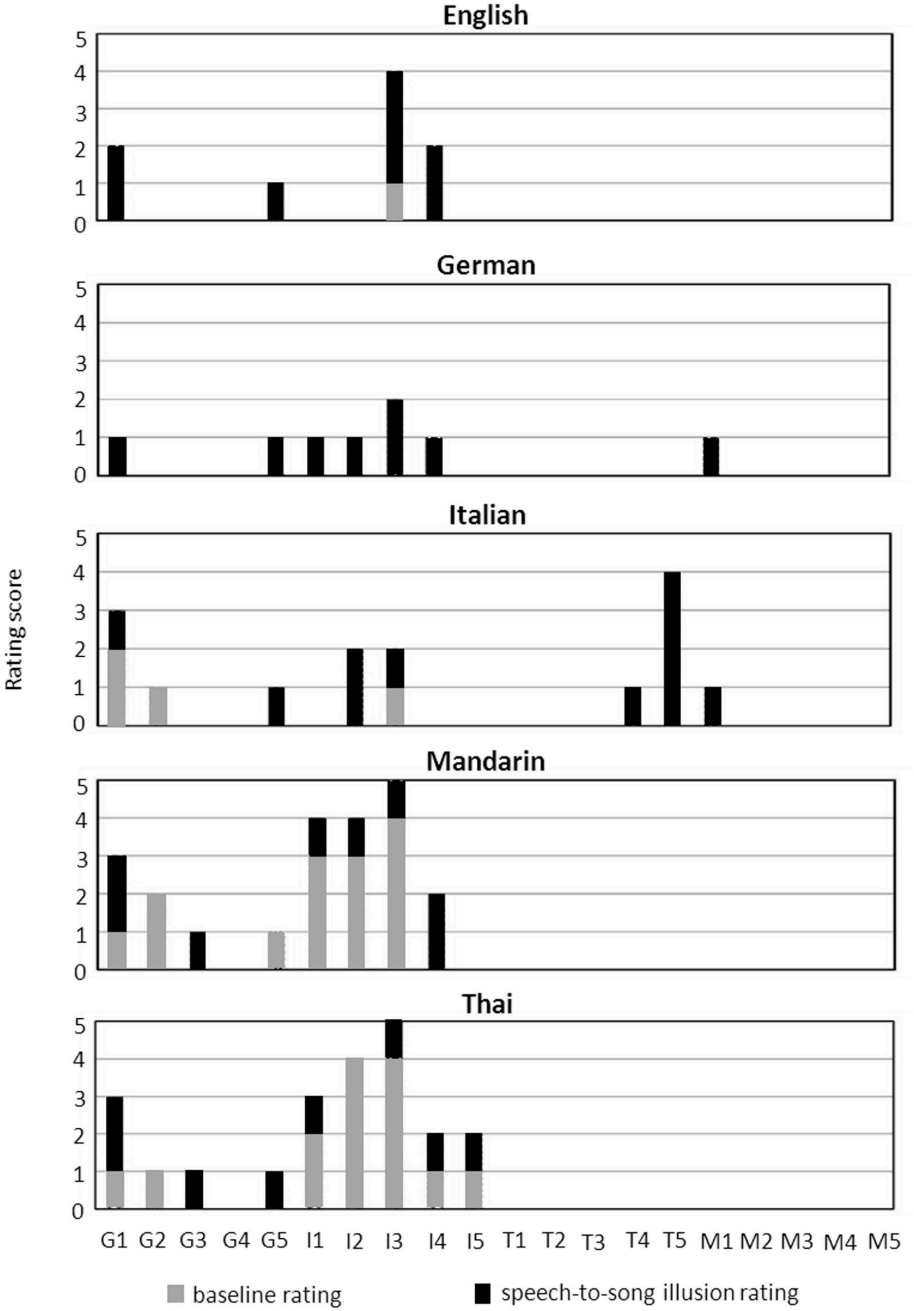
**Rating scores in baseline phase and speech-to-song illusion rating for individual participants**. Data are presented for target excerpts in each stimulus language. Individual speakers coded by native language as follows: G, German; I, Italian; T, Thai; M, Mandarin. Absent bars indicate zero ratings/zero change in rating.

Speech-to-song illusion effects are summarized for each stimulus language across the combined participant group in Table 2, Figure 2; and for each native speaker group across stimulus languages in Table 3, Figure 3. The differential effects of candidate predictor variables on the speech-to-song illusion in bivariate and multivariate regression analyses are summarized in Tables 4, 5, respectively. Individual participant rating data are presented in Figure 4.

**Table 2:** **Mean speech-to-song effect rating scores for target stimuli in each language**.

**Stimulus language**	**Experiment phase**	**Mean (SD) rating**	**Median (IQR) rating**	**Mean rating change**	**Test–Baseline comparison**	
					**Z score**	***p*-value**
English	Baseline	0.05 (0.22)	0 (0)	0.40	1.84	0.066
	Test	0.45 (1.05)	0 (0)			
German	Baseline	0 (0)	0 (0)	0.40	2.53	0.011
	Test	0.40 (0.60)	0 (1)			
Italian	Baseline	0.20 (0.52)	0 (0)	0.55	2.46	0.014
	Test	0.75 (1.16)	0 (1)			
Mandarin	Baseline	0.70 (1.26)	0 (1)	0.40	2.27	0.023
	Test	1.10 (1.65)	0 (2)			
Thai	Baseline	0.70 (1.26)	0 (1)	0.40	2.53	0.011
	Test	1.10 (1.55)	0 (2)			

*Results for the combined participant group are shown. The mean rating change refers to the comparison of ratings in baseline and test phases of the experiment; these phases were compared using Wilcoxon Signed Rank tests. IQR, inter-quartile range; SD, standard deviation*.

**Table 3:** **Mean speech-to-song effect rating scores for each native language participant group**.

**Participant language group**		**Experiment phase**	**Mean (SD) rating**	**Median (IQR) rating**	**Mean rating change**	**Test–Baseline comparison**	
						**Z score**	***p*-value**
Non-tonal	German	Baseline	0.36 (0.64)	0 (1)	0.56	3.071	0.002
		Test	0.92 (1)	1 (1)			
	Italian	Baseline	0.96 (1.46)	0 (2)	0.88	3.660	< 0.001
		Test	1.84 (1.70)	2 (4)			
Tonal	Mandarin	Baseline	0 (0)	0 (0)	0.08	1.414	0.157
		Test	0.08 (0.28)	0 (0)			
	Thai	Baseline	0 (0)	0 (0)	0.20	1.342	0.180
		Test	0.2 (0.82)	0 (0)			

*Participant groups are here classified according to their native language. The mean rating change refers to the comparison of ratings in baseline and test phases of the experiment, across stimulus languages; experimental phases were compared using Wilcoxon Signed Rank tests. IQR, inter-quartile range; SD, standard deviation*.

**Table 4:** **Bivariate associations between predictor variables and speech-to-song effect**.

**Predictor variables**	**Speech-to-song responses**	**No speech-to-song responses**	**Statistical test**	**Statistical values**
Number/% responses in subgroup	31	69	–	–
Age (years): Median (IQR)	28.8 (3.8)	29.6 (9.3)	Ranksum	*Z* = 1.18, *p* = 0.24, *r* = 0.12
Gender: No. (%) male	16 (52)	19 (28)	Chi-square	$\chi_{\left(1\right)}^{2}=$ 5.45, *p* = 0.02
Education (years): Median (IQR)	19 (2.0)	20 (6.0)	Ranksum	*Z* = 0.94, *p* = 0.35, *r* = 0.09
Formal music training (years): Median (IQR)	5(6)	4(9)	Ranksum	*Z* = −1.27, *p* = 0.20, *r* = −0.13
Native language speaker group: No. (%) non-tonal	27 (87)	23 (33)	Fisher's exact	*p* < 0.001
Unable to understand stimulus language: No. (%)	23 (74)	37 (54)	Chi-square	$\chi_{\left(1\right)}^{2}$ = 3.77, *p* = 0.052
Language stimuli: No. (%)				
• Thai	7 (23)	13 (19)	Fisher's exact	*p* = 0.81
• Mandarin	6 (19)	14 (20)		
• German	7 (23)	13 (19)		
• Italian	7 (23)	13 (19)		
• English	4 (13)	16 (23)		

*Data indicate numbers (%) of responses in each category (any speech-to-song effect/no speech-to-song effect; see text) unless otherwise indicated. IQR, inter-quartile range*.

**Table 5:** **Multivariate associations between predictor variables and speech-to-song effect**.

**Predictor variables**	**Multiple logit regression with robust standard error**			**Multiple logit regression with random effects**		
	**OR (SE)**	**95% CI**	***P*-value**	**OR (SE)**	**95% CI**	***p*-value**
Gender : Male (vs. female)	1.6 (1.4)	0.3–8.5	0.562	2.6 (3.5)	0.2–35.7	0.474
Native language: Non-tonal (vs. tonal)	13.7 (10.4)	3.1–60.9	0.001	52.5 (78.2)	2.8–971	0.008
Understands stimulus language: Unable (vs. able)	3.4 (1.6)	1.4–8.5	0.009	7.4 (6.0)	1.5–36.4	0.013

*Results are based on entering candidate predictors from a bivariate analysis (see Table 4) into multiple logit regression analyses with robust standard error and random effects. CI, confidence interval; OR, odds ratio; SE, standard error*.

Considering firstly the participant group as a whole and comparing stimulus languages (Table 2, Figure 2), tonal and non-tonal languages were perceived as comparably “song-like” in the baseline phase (Wilcoxon Signed Rank *Z* = −1.31, *p* = 0.19). As indexed by significantly higher mean ratings of target stimuli in the test compared with the baseline phase, there was evidence of a speech-to-song effect in all languages except English (for which a trend to significance was evident: *p* = 0.066). To explore the strength of association between song-like perception at baseline and the magnitude of any induced speech-to-song effect, the song rating score of each target stimulus in the baseline phase was compared to the difference in scores between baseline and test phases for that stimulus for each participant, using two-tailed spearman correlation over the combined participant group. The extent to which individual participants rated target stimuli as “song-like” at baseline was significantly positively correlated with the magnitude of the speech-to-song effect on those stimuli in the test phase [*r*_s(100)_ = 0.37, *p* < 0.001, two-tailed]. The strength of the speech-to-song effect across the participant group did not differ between non-tonal and tonal language stimuli (Wilcoxon Signed Rank *Z* = −0.97, *p* = 0.35). In participants reporting an increase in song rating between baseline and test phases for any of the target stimuli (i.e., any speech-to-song effect), this effect was always reported during presentation of the target excerpt.

Considering next the effect of listener's native language across stimulus languages, participant groups exhibited differential speech-to-song effects according to native language tonality (Tables 3–5, Figure 3). Non-tonal language native speakers rated languages as overall significantly more song-like at baseline than did tonal language native speakers (Mann-Whitney *U* = 825, *r* = −0.45, *p* = < 0.001; Table 3). Non-tonal language but not tonal language native speakers showed significant speech-to-song-effects (German, Wilcoxon Signed Rank *Z* = 3.07, *p* = 0.002; Italian, Wilcoxon Signed Rank *Z* = 3.66, *p* < 0.001): this was indexed both by increased group rating scores in the test compared with the baseline phase for non-tonal but not tonal language speakers (Table 3) and the higher proportion of instances of the effect attributed to non-tonal language speakers (Table 4). The overall increase in song ratings in the test phase compared with the baseline phase (the overall speech-to-song illusion rating) was significantly greater for non-tonal than tonal native language speakers (tonal vs. non-tonal language group, *U* = 676, *r* = −0.49, *p* < 0.001) whereas illusion ratings for native speakers within a particular language group and for individual native vs. second language (English) did not differ significantly (Italian vs. German speakers, Mann-Whitney *U* = 380, *r* = 0.20, *p* = 0.15; Mandarin vs. Thai speakers, *U* = 311, *r* = −0.006, *p* = 0.97; native language vs. English, Wilcoxon Signed Rank *Z* = −0.17, *p* = 0.86).

In the bivariate analysis assessing the effect of language tonality and other covariates on the speech-to-song illusion (Table 4), significant predictors of the speech-to-song effect were identified as listener's native language group (2-sided Fisher's exact, *p* < 0.001) and male gender [$\chi_{\left(1\right)}^{2}=$ 5.45, *p* = 0.02]. Expressed as relative proportions of participants in each subgroup, non-tonal native language speakers (54%) and male participants (46%) were more likely to experience the speech-to-song effect than tonal native language speakers (8%) and female participants (23%), respectively. In addition, inability to understand the stimulus language was a borderline significant predictor of the speech-to-song effect [$\chi_{\left(1\right)}^{2}$ = 3.77, *p* = 0.052]; no other significant bivariate variables predicting the speech-to-song effect were identified. In a separate post hoc subanalysis assessing the effect of acoustic language stimulus characteristics (duration of spoken source passage, duration of excerpt, mean intra-syllable fundamental frequency change, and inter-syllable duration change), no acoustic stimulus parameters predicted the effect (Table S1). Bivariate associations of native language tonality and inability to understand the stimulus language were confirmed in multivariate logit regression analyses both with robust standard error and taking random effects into account (Table 5): in particular, accounting for random effects the odds of showing the speech-to-song illusion effect were 52-fold higher in non-tonal than tonal native language speakers. Gender was a significant predictor in the bivariate analysis but became non-significant in the multivariate logit regression analysis: this indicates that the bivariate effect of gender was attributable to a covarying factor (here, native language tonality).

Individual participant rating data (Figure 4) indicated variation in ratings between individuals and stimulus languages but (as predicted from the group data) a clear disparity between tonal vs. non-tonal native language groups, speech-to-song effects being largely absent in tonal native language speakers; of the four speech-to-song responses recorded for the tonal native language group, three were reported for the Italian stimulus.

## Discussion

Here we have shown that the speech-to-song illusion is reduced in native speakers of tonal languages relative to non-tonal language speakers. The speech-to-song illusion effect was only reported for stimuli used to induce the illusion, consistent with a specific perceptual transformation; the effect was not dependent on language stimulus factors. The overall strength of the speech-to-song effect here was relatively weak. These observations are in line with previous data on cross-language speech-to-song effects (Margulis et al., 2015). In addition, the illusion was significantly enhanced by reduced language comprehension; consistent with this and also with previous work (Margulis et al., 2015), English (the only language in which all participants were proficient) was the only language not to show a significant speech-to-song effect for the combined participant group.

We did not find an effect from certain potentially relevant modulators of the speech-to-song illusion. The participant language groups in this study had comparable exposure to formal musical training. While absolute pitch is substantially more frequent among tonal than non-tonal language speakers (Deutsch et al., 2009) and could potentially modulate propensity to experience the speech-to-song illusion (Falk et al., 2014), this factor can be discounted here as none of our participants possessed absolute pitch. It seems unlikely that the speech-to-song effect was attenuated by greater linguistic competence of tonal language native speakers: if the effect were primarily driven by greater difficulty of articulatory recoding of the stimulus language as previously suggested in a comparison between non-tonal languages (Margulis et al., 2015), then one might have expected non-tonal language native speakers to show an enhanced effect for tonal vs. non-native non-tonal languages. Information is limited concerning any effect of gender on the speech-to-song illusion: though we did not find an independent effect of gender in this study, the gender distribution between tonal and non-tonal native language groups was skewed (Table 1). Moreover, gender effects on pitch pattern perception are likely a priori (Everhart et al., 2006) but these may be relatively subtle and may therefore have escaped detection here. It is also possible that a wider range of acoustic language parameters might capture an effect, though noteworthy that previous detailed analyses have similarly found no effect from acoustic properties *per se* (Margulis et al., 2015).

In our participant group, a propensity to rate stimuli as song-like at baseline predicted a stronger speech-to-song illusion over all language stimuli. This observation may hold a clue to the cognitive mechanism whereby speech-to-song perceptual transformations are attenuated in tonal language speakers relative to non-tonal language speakers. Implicitly encoded musical structural “rules” have been shown to influence perceptual judgments during speech-to-song transformations (Vanden Bosch der Nederlanden et al., 2015). We interpret the present data as further evidence that native language tonality may determine the extent to which prosodic pitch structures are perceived inherently as conforming to musical or lexical “melody” (Bidelman and Lee, 2015). Such an inherent “bias” is supported by the differential baseline song ratings of language stimuli by tonal and non-tonal language native speakers here; this bias may in turn influence how easily any additional perceptual transformation of speech toward music (i.e., the speech-to-song illusion) can occur. If tonal language native speakers are predisposed to code pitch patterns as linguistic based on past experience, such listeners may be less likely to recode pitch patterns as musical (less likely to experience the speech-to-song illusion) than non-tonal language speakers. This interpretation is in line with behavioral and functional neuroimaging evidence for preferential “templating” of pitch patterns as linguistic in tonal language speakers (Bidelman and Lee, 2015).

On the other hand, inability to understand the speech stream as a verbal message might facilitate processing of prosodic features as music during induction of the speech-to-song illusion (Falk et al., 2014; Margulis et al., 2015): the present data comparing first and second languages in these bilingual participants suggests that differential linguistic competence (for languages that the listener understands) may not modulate the illusion, though ideally this would be explored in a larger sample covering a wider range of language combinations and linguistic ability. It may be that the robustness of cognitive mechanisms for maintaining perceptual speech-song “boundaries” is an important determinant of illusion strength. Previous functional neuroimaging work suggests that any native language tonality effect on phoneme perception is modulated by specific language experience (Zatorre and Gandour, 2008). Inspection of the individual data (Figure 4) suggests that weaker interactions may operate between particular native language—stimulus language combinations and may hold further clues to the mechanism of the speech-to-song illusion. Disambiguation of language experience from language understanding is challenging, since greater understanding of a language in general necessarily entails greater experience with its tonal and prosodic structures. Pending further data with a wider repertoire of language competencies, interpretation must be qualified due to the small sample sizes here.

We regard these results as preliminary, providing a basic proof-of-principle for a native language tonality effect and a rationale for further systematic work to substantiate the findings. Larger cohorts sampling a wider range of tonal and non-tonal languages will be required to corroborate, extend and establish the reliability of our observations. Furthermore, given the relatively small numbers of participants and language samples, this study will have been under-powered to detect more subtle, potentially relevant interactions (for example, between stimulus language and language familiarity or the effect of gender): apparently “negative” associations here should, therefore be interpreted as provisional. The effects here might be amplified by systematic selection of speech excerpts previously shown robustly to induce the speech-to-song illusion (Tierney et al., 2013) and by use of a signal detection framework that would capture any “false alarms.” Larger samples with more rigorous control of non-native language competence might also allow the impact of particular factors (such as articulatory recoding difficulty) to be assessed directly. Manipulation of stimulus prosodic structure could allow a direct test of our hypothesis concerning the role of perceived language musicality as well as assessing any role of metrical and other properties that vary between languages and might contribute to generation of the speech-to-song illusion. With this in mind, it would also be of particular interest to investigate the speech-to-song illusion in specific populations, such as absolute pitch possessors, individuals with congenital amusia and those with strategic brain damage involving pitch processing networks. The effect of musical training on the illusion (though not identified as a predictor here) remains to be fully defined (Falk et al., 2014) and might be explored in larger musician cohorts and assessing different kinds of musical expertise. It may also be informative to study speech-to-song effects longitudinally during second language learning, to assess any modulation by enhanced language experience and familiarity. Ultimately, there is a need to establish neural substrates using correlative structural and functional brain imaging: it is practical to investigate the speech-to-song illusion using fMRI (Tierney et al., 2013) and a study of tonal—nontonal bilingual speakers may be particularly pertinent (Zatorre and Gandour, 2008), while acknowledging that the perceptual organization of the language system in blingual individals may well not be “typical” of the wider population.

## Author contributions

All authors fulfill criteria for authorship as listed at: http://journal.frontiersin.org/journal/psychology/section/auditory-cognitive-neuroscience#author-guidelines. KJ, JW made substantial contributions to the conception and design of the work. KJ, RS, and MF were involved in data acquisition and analysis. All authors were involved in drafting or revising the paper critically for important intellectual content, gave approval for the final version to be published and agree to be accountable in ensuring that questions related to the accuracy or integrity of any part of the work are appropriately investigated and resolved.

## Funding

The Dementia Research Centre is supported by Alzheimer's Research UK, the Brain Research Trust and the Wolfson Foundation. This work was funded by the Wellcome Trust, the UK Medical Research Council and the NIHR Queen Square Dementia Biomedical Research Unit. MF is funded by Becas Chile, Conicyt-PCHA/2012/72130281. JW is supported by a Wellcome Trust Senior Clinical Fellowship (Grant No 091673/Z/10/Z).

### Conflict of interest statement

The authors declare that the research was conducted in the absence of any commercial or financial relationships that could be construed as a potential conflict of interest.
