# Supplementary material for: The Speech-to-Song Illusion Is Reduced in Speakers of Tonal (vs. Non-Tonal) Languages
Source: Front Psychol. 2016 May 9;7:662. doi: 10.3389/fpsyg.2016.00662 (PMC4860502; doi:10.3389/fpsyg.2016.00662)
Supplement: Supplementary file 1 [file Table1.DOCX]

**Supplementary Material: The speech-to-song illusion is reduced in speakers of tonal (versus non-tonal) languages, by K Jaisin et al**

Two acoustic characteristics were derived for all stimuli: fundamental frequency variability and temporal variability. The onset and offset of each syllable were manually marked on TextGrids using Praat (Boersma & Weenink, 2015). Fundamental frequency was measured as mean *f*_0_ at the syllable level, from Pitch objects generated using the two-pass method (Hirst, 2011; de Looze & Hirst, 2008). Both fundamental frequency variability and temporal variability were quantified as median absolute deviations (MAD), which were calculated in R (R Core Team, 2013).

**Table S1.** Summary of stimulus characteristics

| **Languages** | **Duration of spoken source passage** (sec) | **Duration of excerpt**  (sec) | **MAD intra-syllable fundamental frequency change** (Hz) | **MAD intra-syllable duration change**  (msec) |
| --- | --- | --- | --- | --- |
| Thai | 10.4 | 1.8 | 39.12 | 47.27 |
| Mandarin | 10.8 | 2.8 | 26.33 | 13.63 |
| German | 13.6 | 2.2 | 18.43 | 83.76 |
| Italian | 10.3 | 2.6 | 27.73 | 58.36 |
| English | 15.1 | 2.6 | 19.15 | 84.85 |
| Thai foil | 10.6 | - | - | - |
| German foil | 13.5 | - | - | - |
| English foil | 15.3 | - | - | - |

Acoustic parameters were not derived for foil stimuli. MAD; median absolute deviation. None of these parameters was found to be significantly associated with the speech-to-song illusion effect in a post hoc bivariate analysis (MAD intra-syllable fundamental frequency change; Mann-Whitney *U* = 1009.5, *r*= -0.046, *p* =0.648, MAD intra-syllable duration change, *U* = 989.5, *r*= -0.060, *p* =0.543). See main text for further explanation.

**Supplementary references**

Boersma, Paul & Weenink, David (2015). Praat: doing phonetics by computer [Computer program]. Version 6.0.08, retrieved 5 December 2015 from http://www.praat.org/

Hirst, D. J. (2011). The analysis by synthesis of speech melody: from data to models. Journal of speech Sciences, 1(1), 55-83.

De Looze, C., & Hirst, D. J. (2008, May). Detecting changes in key and range for the automatic modelling and coding of intonation. In Proceedings from Speech Prosody.

R Core Team (2013). R: A language and environment for statistical computing. R Foundation for Statistical Computing, Vienna, Austria. URL http://www.R-project.org/.
