# Supplementary material for: The Speech-to-Song Illusion Is Reduced in Speakers of Tonal (vs. Non-Tonal) Languages
Source: Front Psychol. 2016 May 9;7:662. doi: 10.3389/fpsyg.2016.00662 (PMC4860502; doi:10.3389/fpsyg.2016.00662)
Supplement: Supplementary file 2 [file Table2.DOCX]

Warren_Audio_1 English: foil

Warren_Audio_2 English: induction phase

Warren_Audio_3 English: real singing

Warren_Audio_4 English: stimuli

Warren_Audio_5 German: foil

Warren_Audio_6 German: induction phase

Warren_Audio_7 German: stimuli

Warren_Audio_8 Italian: induction phase

Warren_Audio_9 Italian: stimuli

Warren_Audio_10 Mandarin: induction phase

Warren_Audio_11 Mandarin: stimuli

Warren_Audio_12 Thai: foil

Warren_Audio_13 Thai: induction phase

Warren_Audio_14 Thai: stimuli
